# Intraoperative Ultrasound Shear-Wave Elastography in Focal Cortical Dysplasia Surgery

**DOI:** 10.3390/jcm10051049

**Published:** 2021-03-03

**Authors:** Bertrand Mathon, Stéphane Clemenceau, Alexandre Carpentier

**Affiliations:** 1 Department of Neurosurgery, La Pitié-Salpêtrière University Hospital, Assistance Publique-Hôpitaux de Paris, 75013 Paris, France; stephane.clemenceau@aphp.fr (S.C.); alexandre.carpentier@aphp.fr (A.C.); 2 Faculty of Medicine, Sorbonne University, 75005 Paris, France; 3 Paris Brain Institute (ICM, INSERM, UMRS 1127, CNRS, UMR 7225), 75013 Paris, France

**Keywords:** epilepsy surgery, drug-resistant epilepsy, neurosurgery, shear-wave elastography, real-time guidance, epileptogenic zone, brain surgery, focal epilepsy, ultrasound, seizure outcome

## Abstract

Previous studies reported interest in intraoperative shear-wave elastography (SWE) guidance for brain-tumor and epilepsy surgeries. Focal cortical dysplasia (FCD) surgery is one of the most appropriate indications for using SWE guidance. The aim of this study was to evaluate the efficacy of ultrasound SWE techniques for the intraoperative detection of FCDs. We retrospectively analyzed data from 18 adult patients with drug-resistant epilepsy associated with FCD who had undergone SWE-guided surgery. Conventional B-mode images detected FCD in 2 patients (11.1%), while SWE detected FCD in 14 patients (77.8%). The stiffness ratios between MRI-positive and -negative cases were significantly different (3.6 ± 0.4 vs. 2.2 ± 0.6, respectively; *p* < 0.001). FCDs were significantly more frequently detected by interoperative SWE in women (OR 4.7, 95% CI (1.7–12.7); *p* = 0.004) and in patients in whom FCD was visible on magnetic resonance imaging (MRI; OR 2.3, 95% CI (1.3–4.3); *p* = 0.04). At 1 year after surgery and at last follow-up (mean = 21 months), seizure outcome was good (International League Against Epilepsy (ILAE) Class 1 or 2) in 72.2% and 55.6% of patients, respectively. Despite some limitations, our study highlighted the potential of SWE as an intraoperative tool to detect FCD. Future technical developments should allow for optimizing intraoperative surgical-cavity evaluation from the perspective of complete FCD resection. Interobserver reliability of SWE measurements should also be assessed by further studies.

## 1. Introduction

Epilepsy globally affects at least 50 million people [1]. Focal cortical dysplasia (FCD) is responsible for 30% to 50% of drug-resistant epilepsies [2]. FCDs are characterized by deranged neurons in white matter, dyslamination, and abnormal balloon cells, and thereby have stiffer consistence than that of the surrounding healthy parenchyma [3]. A complete surgical resection of FCD leads to postsurgical seizure control in up to 80% of patients [4]. FCDs cannot be seen by the naked eye or through a surgical microscope, and are mostly not detected by 3.0 T magnetic resonance imaging (MRI). The accurate intraoperative detection of FCD is thus extremely challenging for neurosurgeons.

Shear-wave elastography (SWE) is an ultrasound imaging technique that analyzes viscoelastic tissue characteristics, thus generating qualitative and quantitative evaluations of elasticity values [5]. SWE allows for the real-time intraoperative cartography of the brain parenchyma by discriminating healthy from lesional tissue [6]. Since the first report of the use of SWE guidance in brain surgery in 2014 [7], several authors examined its contribution for brain-tumor surgery [8,9,10]. In 2019, we assessed interest in SWE for epilepsy surgery in a heterogeneous case series of 28 patients with epileptogenic lesions (FCDs, dysembrioplastic neuroepithelial tumors, cavernomas, gangliogliomas, and post-traumatic lesions) [11]. In this preliminary study, we hypothesize that SWE is particularly appropriate for FCD patients for the reasons mentioned above. The aim of this study is to evaluate the efficacy of ultrasound SWE techniques for the intraoperative detection of FCDs.

## 2. Experimental Section

### 2.1. Patients

We retrospectively analyzed all adult patients with refractory epilepsy associated with FCD who had undergone SWE-guided surgery at our institution. Data from presurgical evaluations were analyzed to determine whether an epileptogenic zone could be clearly identified. Intracranial electroencephalographic (iEEG) recordings were performed only in MRI-negative patients. All patients had a histopathological diagnosis of FCD.

Age, gender, seizure characteristics, intraoperative B-mode, and elastography characteristics were prospectively collected, as well as preoperative and postoperative (at 12 months) neurological examinations, brain MRI, and postoperative histology. All patients were followed for at least 1 year. Seizure outcome was evaluated using the International League Against Epilepsy (ILAE) classification [12]. Good postoperative outcome was defined by ILAE Classes 1 and 2.

### 2.2. Intraoperative B-Mode and SWE Image Acquisition

All surgical procedures were performed under general anesthesia. The patients’ head was fixed in a Mayfield holder. In all patients, neuronavigation (Stealthstation™ s7, Medtronic, Minneapolis MN, USA) based on preoperative MRI was performed to localize the lesion site.

Intraoperatively, ultrasound images and stiffness maps were acquired with an ultrafast ultrasonic device (Aixplorer^®^, Supersonic Imagine, Aix-en-Provence, France) using the same probe. The ultrasound-image and stiffness-map acquisition protocol was described previously [11]. Briefly, ultrasound parameters were adjusted before FCD analysis. B-mode images and stiffness maps were recorded after dural opening in order to monitor dysplastic-area resection. FCD was analyzed on both axes. The axis with the best view of the dysplastic area was chosen to provide the stiffness-map image. Stiffness was color-coded in kilopascals (kPa) and merged with the B-mode image. Deep blue coloration represented the softest elasticity, and dark red represented the hardest. The operator then chose a region of interest (Qbox©) both in the dysplasia and in the healthy cortex in order to measure tissue stiffness. Stiffness was quantified by Young’s modulus (mean +/− standard deviation). A ratio (Qbox ratio©) was obtained by dividing the lesion stiffness by normal brain stiffness. We considered the positivity of the dysplastic area detection if this ratio was higher than 2. Digital Imaging and Communication in Medicine (DICOM) images were generated for each patient. All dysplastic areas and healthy brain elasticities were analyzed by the same operator. At the end of the surgery, a final ultrasound imaging was recorded with B-mode and SWE to evaluate the completeness of the resection.

### 2.3. Statistical Analysis

Results expressed as number (%) were compared with a Χ^2^ test; continuous variables expressed as mean +/− standard deviation were compared with the Student’s *t*-test. Patients’ demographic, clinical, and radiological data were tested in univariable analysis for association with obtaining the intraoperative detection of FCD by SWE techniques; *p* < 0.05 defined statistical significance. Analyses were performed with IBM SPSS Statistics version 22 software (IBM Corporation, Armonk, NY, USA).

### 2.4. Ethics

The database was subject to a declaration to the CNIL, the French Data Protection Authority, under number 2214386. In accordance with the ethical standards of our hospital’s institutional review board and French law, the need for signed patient consent was waived. The article was designed and written in accordance with STROBE guidelines.

## 3. Results

### 3.1. Patient Demographics

We analyzed FCD surgeries using intraoperative SWE guidance in 18 patients with refractory focal epilepsy. Mean age at the time of surgery was 25.7 ± 7.3 years (18–43). Sex ratio (F/M) was 1.6. Mean age at epilepsy onset was 13.9 ± 5.0 years (7–23). Thirteen (72.2%) patients consumed at least 3 antiepileptic drugs at the moment of surgery. Ten of the patients (55.6%) had normal MRI exploration (nonlesional epilepsy) and required prolonged continuous 24 h video-intra-cranial electroencephalogram (iEEG) records to localize the epileptogenic zone. Eleven patients (61.1%) were operated on frontal-lobe epilepsy, 5 (27.8%) on parietal-lobe epilepsy, 1 (5.6%) on occipital-lobe epilepsy, and 1 (5.6%) on temporal-lobe epilepsy. The epileptogenic zone was located in the left hemisphere in 11 patients (61.1%).

### 3.2. SWE Findings and Lesion Characteristics

**Figure 1:**
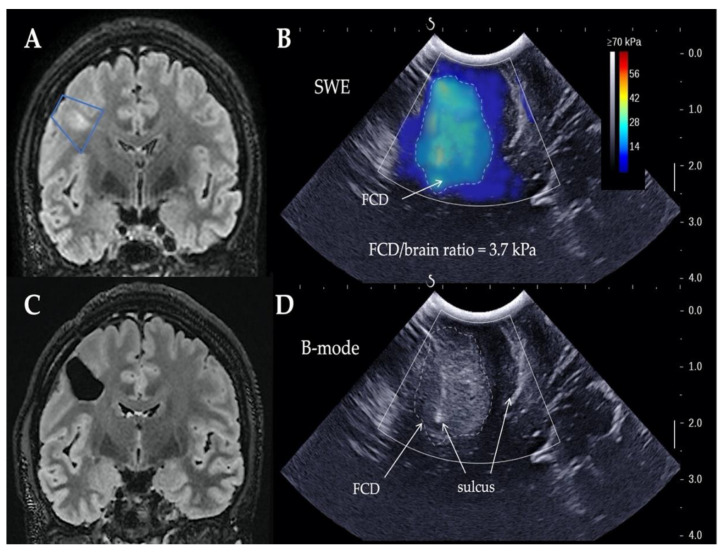
(**A**) Preoperative and (**C**) postoperative magnetic resonance imaging (MRI) images (T2-weighted FLAIR, coronal views) in patient with MRI-positive right frontal focal cortical dysplasia (FCD). Blue trapezium: ultrasound imaging plan. (**B**) Intraoperative elastography and (**D**) ultrasound B-mode images. Dimensions of ultrasound images: 38.4 mm wide and 40.0 mm deep. (**B**) Zone of increased stiffness (dotted line) highlighted FCD, and (**D**) B-mode image showed abnormal hyperechogenicity. SWE, shear wave elastography.

**Figure 2:**
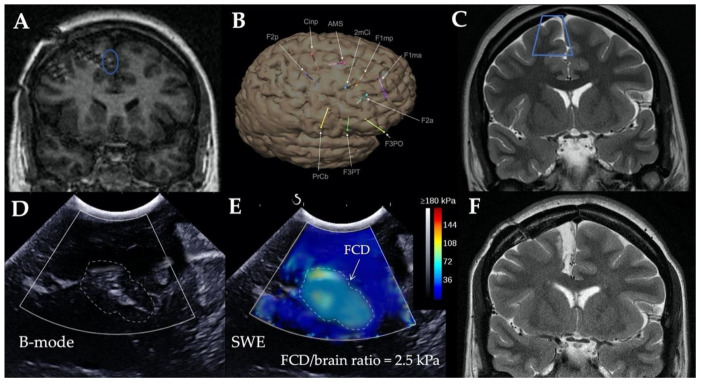
Preoperative MRI images ((**A**) T1- and (**C**) T2-weighted, coronal views), in a patient with right mesiofrontal cryptogenic epilepsy. Intracranial electroencephalogram (EEG) recordings located epileptogenic zone around deepest contact of the F1mp electrode ((**A**), blue circle, and (**B**)). (**C**) Blue trapezium: ultrasound imaging plan. (**D**) Intraoperative ultrasound B-mode images did not find any abnormality, while (**E**) elastography images detected small heterogeneous area of stiffer parenchyma consistent with focal cortical dysplasia. (**F**) Right medial frontal surgical resection allowed for patient to achieve seizure freedom. FCD, focal cortical dysplasia; SWE, shear wave elastography.

All patients underwent intraoperative SWE evaluation prior to FCD resection. As the two examples below illustrate (Figure 1 and Figure 2), echogenicity, stiffness maps, stiffness values, and ratios were obtained for all patients. The mean duration of SWE acquisition and interpretation was 7 ± 2 min (range, 4–10).

B-mode images displayed echogenicity differences between dysplastic area and healthy parenchyma in 2 patients (11.1%), while SWE detected a >2 stiffness ratio in 14 patients (77.8%; *p* = 0.42, Table 1). The 8 cases (100%) of FCD visible on MRI were detected by SWE, while 2 cases (25%) were visualized by B-mode images. Concerning nonlesional epilepsy, none of the 10 MRI-negative FCD was visualized by B-mode images, while 6 cases (60%) were detected by SWE. Stiffness ratios between MRI-positive and -negative cases were significantly different (3.6 ± 0.4 vs. 2.2 ± 0.6 respectively; *p* < 0.001); effect size, measured using Cohen’s d, was medium (0.66). In all cases, SWE was not helpful to assess the completeness of resection.

### 3.3. Factors Asscoiated with FCD Detection

Comparisons between patients with SWE-detected and -nondetected FCD are reported in Table 2. FCDs were significantly more frequently detected by interoperative SWE in women (odds ratio 4.7, 95% confidence interval (1.7–12.7); *p* = 0.004) and in patients with MRI-positive FCD (OR 2.3, 95% CI (1.3–4.3); *p* = 0.04).

### 3.4. Postoperative Outcomes

In MRI-positive patients, postoperative MRI showed a complete resection of the FCD in 7 out of 8 cases (87.5%). Three patients (16.7%) experienced transient motor or sensitive postoperative deficit that fully recovered in weeks. No infection related to surgery were observed.

**Figure 3:**
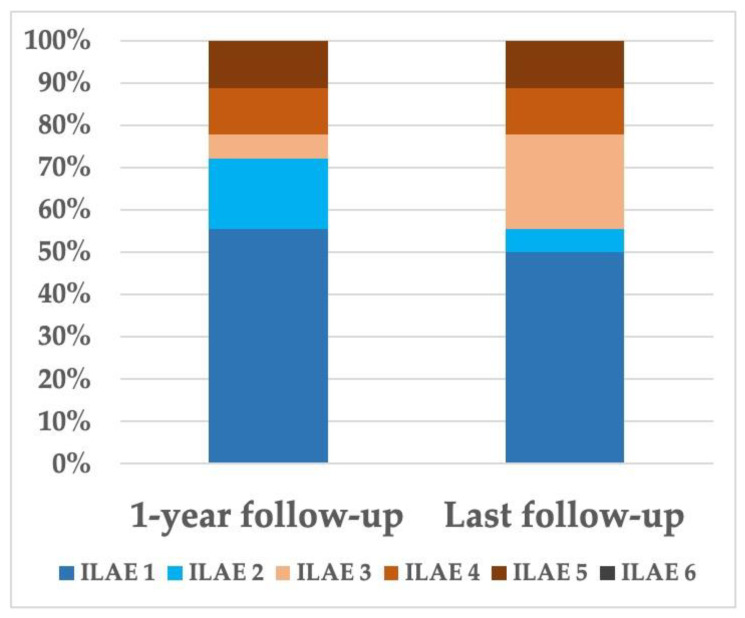
Postoperative seizure outcomes. International League Against Epilepsy (ILAE) classification of patients during postoperative follow-up. Good postoperative seizure outcome was defined in our study by ILAE Classes 1 and 2.

At 1 year after surgery and at last follow-up (mean = 21 months, range 12–40), seizure outcome was good in 13 (72.2%) and 10 (55.6%) patients, respectively (Figure 3). Univariable analyses (Table 3) identified patients with a favorable seizure outcome at 1 year as having only more frequent complete FCD resection (*p* = 0.005).

## 4. Discussion

Our study analyzed the intraoperative B-mode and SWE characteristics of FCDs. We demonstrated that SWE has high sensitivity in intraoperative FCD detection, confirming the capacity of advanced ultrasound techniques as a reliable image guidance technique for the detection of epileptogenic lesions. Logically, FCDs were more frequently detected by interoperative SWE in patients in with MRI-positive FCD. Because of the low statistical power of our study, we were unable to demonstrate the influence of intraoperative SWE guidance on improving postoperative seizure outcome.

### 4.1. Shear-Wave Elastography Feedback and Interobserver Reproducibility

On the basis of these results and our experience, we propose a summary of application for SWE in FCD surgery (Table 4). Previous studies on intraoperative elastography in epilepsy surgery already discussed and debated SWE-related advantages and limitations [11,13]. Nevertheless, the interobserver reproducibility of intraoperative elastography findings is a crucial issue and should be discussed in detail. As elastography is an operator-dependent technique, differences in experience between neurosurgeons may result in distinct findings. Thus, variation in interobserver reliability in elastography currently limits its applicability.

Strain elastography that utilizes external stimuli is the most difficult elastography technique to reproduce. Stiffness quantifications with this method are subjective since the same level of compression is tricky to reproduce between different operators [14]. Moreover, the excess of compression applied by the operator usually produces artifacts that result in erroneous measurements [14,15]. The choice of regions of interest can also be subject to variability [16].

Contrary to strain elastography, SWE does not necessitate extrinsic stress, and thereby reduces intraobserver variability. In 5 patients of their cohort, Chauvet et al. obtained an excellent intraclass correlation coefficient (ICC = 0.99) for intraobserver repetition and reproduction. Regarding interobserver reproducibility, they reported good ICC for both repetition (ICC = 0.96) and reproduction (ICC = 0.93) [8]. In a pediatric cohort with healthy volunteers and patients with intracranial pathology, El-Ali et al. reported that SWE measurements were reproducible in both groups [17]. Further studies assessing interoperator reliability by ICC or Cohen’s kappa are required to answer this question.

In our study, stiffness maps were recorded only after dural opening. Nonetheless, further works could study differences between SWE conducted on the same region of interest before and after dural opening. Indeed, it would be of interest to know if a cerebrospinal-fluid leak affects tissue elasticity. Theoretically, changes in brain pulsatility could slightly modify SWE measurements, but should not affect the stiffness ratio.

### 4.2. SWE Safety

No side effects related to intraoperative ultrasonography were reported in our series. In particular, SWE acquisition and analysis took little more than 10 min and were not associated with an increased postoperative-infection rate. SWE has been used in other organ surgeries for several years now without any associated adverse events [18], and is validated by international recommendations for adult patients [16,19,20,21,22,23]. Although there is no evidence for its safety on infants and children [24], preliminary animal data suggest that SWE in the newborn brain is free of harm [25,26]. A very recent work demonstrated that when the brain of a newborn mouse was exposed to elastography for at least a 10 min period, the gene expression of synaptic function was temporarily altered, but learning and memory were preserved in adulthood [27].

In conclusion, to date, no risks were identified in studies achieved on the basis of the “as low as reasonably achievable” (ALARA) rule [28].

### 4.3. SWE in the Intraoperative Ultrasound Technique Armamentarium

Although previous studies suggested interest in B-mode to detect FCDs [29,30,31], B-mode enabled us to identify the dysplastic area in only a minority of cases. Our findings and the literature showed that SWE is more sensitive than B-mode is for the intraoperative detection of FCDs.

Strain elastography is an alternative elastography method that is relatively insensitive to brain anisotropy, while shear waves propagate within a homogeneous parenchyma [14,32,33,34,35]. Some authors argued that, because of these technical differences, strain elastography has higher spatial resolution compared to that of SWE, allowing for better contrast resolution and the definition of FCD components [13]. Nevertheless, whatever the used method, elastography cannot precisely define FCD boundaries.

Lastly, contrast-enhancement ultrasound, which requires microbubble injection with ultrasound delivery, is currently under evaluation, so its use is limited to research works [13].

### 4.4. SWE Compared to Other Intraoperative Tools

Compared to intraoperative conventional neuronavigation systems, SWE is not impacted by brain shift, which can make it difficult to accurately locate FCD within the brain [36]. Recently navigated 3D ultrasound systems could address this major issue [37,38]. Electrocorticography may also help to identify the epileptogenic focus by detecting abnormal electrical activity [39]. However, in deep-seated FCDs, electrocorticography hardly detects paroxysmal activity. Intraoperative MRI improves the degree of FCD resection and postoperative seizure outcome [40], but does not provide a spatial resolution as good as that of preoperative images, and is useless in MRI-negative patients. Given the characteristics of these different intraoperative techniques and our findings, the combination between SWE and neuronavigation is complementary and useful in FCD surgery guidance.

### 4.5. Future Perspectives

In our experience and to date, SWE is not helpful in assessing the completeness of FCD resection. The presence of air, blood clots, or inflammation is associated with artefacts that preclude the reliable assessment of the operative site when looking for residual dysplastic tissue [25]. Future technical developments should be added to rectify this phenomenon.

As stated above, SWE is not suitable for deep-seated epileptogenic lesions (e.g., mesial temporal-lobe epilepsy) because depth reduces shear-wave spread. In the near future, probe miniaturization and shear-wave field enhancement should allow for significant advances for the elastographic exploration of deep-seated lesions [41,42,43].

Overall, with the advancement of computing technologies and ongoing improvements of elastography imaging devices, SWE is expected to resolve current weaknesses of conventional ultrasound. It would definitely be more helpful in accurately discriminating the FCD from the surrounding normal cortex and in precising FCD boundaries [25]. Although the intraoperative use of SWE in epilepsy surgery has only just been established, experimental laboratory work and initial clinical experiences are very hopeful.

### 4.6. Study Limitations

Our study presents some limitations. First, it is a single-center study with a small sample of patients and retrospective design, while data were prospectively acquired. Second, the interobserver reproducibility of the intraoperative ultrasound measurements was not assessed. However, our work confirmed preliminary results showing the potential contribution of SWE to FCD surgery, and paves the way to larger studies. Lastly, we could not correlate cytological and architectural abnormalities of FCD with SWE findings.

## 5. Conclusions

This study underscores the role of SWE as an efficient tool for the intraoperative detection of FCDs. Future technical developments should allow for optimizing intraoperative surgical-cavity evaluation from the perspective of complete FCD resection. Larger multicenter studies are required to confirm these findings and to investigate whether intraoperative SWE guidance can reach better seizure outcomes.

## Figures and Tables

**Table 1:** Comparison between sensitivity of B-mode and SWE mode for intraoperative detection of FCDs.

FCDs	*n*	B-Mode Sensitivity % (*n*)	SWE Sensitivity % (*n*)	FCD/Healthy Brain Stiness Ratio
MRI + FCD	8	25% (2)	100% (8)	3.6 ± 0.4
MRI − FCD	10	0% (0)	60% (6)	2.2 ± 0.6
Total	18	11.1% (2)	77.8% (14)	2.8 ± 0.9

FCD, focal cortical dysplasia; MRI − FCD, MRI-negative focal cortical dysplasia; MRI + FCD, focal cortical dysplasia visible on MRI; SWE, shear-wave elastography.

**Table 2:** Patient and FCD characteristics with comparison according to SWE detection status.

Characteristics	All Patients *n* = 18	FCD Detected by SWE *n* = 14	FCD Not Detected by SWE *n* = 4	*p*-Value
Females	11 (61.1)	11 (100)	0 (0)	**0.004**
Age on surgery day, years	25.7 ± 7.3	26.5 ± 8	22.8 ± 3.3	0.19
Epilepsy history				
Age at epilepsy onset, years	13.9 ± 5	14.4 ± 5.3	12.5 ± 4.4	0.50
3 or more antiepileptic drugs	13 (72.2)	10 (71.4)	3 (75)	0.89
Epileptogenic-zone location				
Left hemisphere	11 (61.1)	9 (64.3)	2 (50)	0.61
Frontal lobe	11 (61.1)	8 (57.1)	3 (75)	0.52
Parietal lobe	5 (27.8)	4 (28.6)	1 (25)	0.89
Temporal lobe	1 (5.6)	1 (7.1)	0 (0)	0.58
Occipital lobe	1 (5.6)	1 (7.1)	0 (0)	0.58
FCD radiological characteristics				
MRI + (iEEG−)	8 (44.4)	8 (57.1)	0 (0)	**0.04**
Intraoperative characteristics				
SWE acquisition duration, min	6.6 ± 1.7	6.4 ± 1.6	7.5 ± 1.9	0.33

iEEG−, no intracranial electroencephalogram performed during presurgical evaluation; MRI + FCD, focal cortical dysplasia visible on MRI; SWE, shear-wave elastography.

**Table 3:** Patient and FCD characteristics with comparison according to seizure outcome.

Characteristic	All Patients *n* = 18	Good Seizure Outcome at 1 Year *n* = 18	*p*-Value	Good Seizure Outcome at Last Follow-Up *n* = 4	*p*-Value
Females	11 (61.1)	9 (62.9)	0.26	7 (70)	0.39
Age on surgery day, years	25.7 ± 7.3	27 ± 8.2	0.07	28.6 ± 8.7	0.053
Epilepsy history					
Age at epilepsy onset, years	13.9 ± 5	15.3 ± 4.9	0.06	15.3 ± 5.6	0.19
3 or more antiepileptic drugs	13 (72.2)	10 (76.9)	0.47	8 (80)	0.41
Epileptogenic-zone location					
Left hemisphere	11 (61.1)	9 (69.2)	0.26	6 (60)	0.91
Frontal lobe	11 (61.1)	7 (53.8)	0.31	5 (50)	0.28
Parietal lobe	5 (27.8)	4 (30.8)	0.65	3 (30)	0.81
Temporal lobe	1 (5.6)	1 (7.7)	0.52	1 (10)	0.36
Occipital lobe	1 (5.6)	1 (7.7)	0.52	1 (10)	0.36
FCD radiological characteristics					
MRI + (iEEG−)	8 (44.4)	7 (53.8)	0.20	6 (60)	0.14
Intraoperative characteristics					
SWE acquisition duration, min	6.6 ± 1.7	6.5 ± 1.8	0.76	6.8 ± 1.9	0.6
FCD detected by B-mode	2 (11.1)	2 (15.4)	0.35	2 (20)	0.18
FCD detected by SWE	14 (77.8)	11 (84.6)	0.26	8 (80)	0.8
Postoperative characteristics					
Complete FCD resection	7/8 (87.5)	7 (100)	**0.005**	6 (100)	0.06

iEEG−, no intracranial electroencephalogram performed during presurgical evaluation; MRI + FCD, focal cortical dysplasia visible on MRI; SWE, shear-wave elastography. Good postoperative outcome defined in our study by ILAE Classes 1 and 2.

**Table 4:** Advantages, disadvantages, and considerations concerning use of SWE for intraoperative detection of focal cortical dysplasia.

Advantages	Disadvantages	Considerations
Real-time imaging technique	Low sensitivity to evaluate completeness of FCD resection	Need to train on easy cases (e.g., meningiomas and high-grade gliomas) before using on FCD patients
High sensitivity for detecting FCDs	Significant learning curve	Need to know limits of SWE technique
Easy and fast to use	Operator-dependent tool	Need to assess interobserver reproducibility
Safe technique (no related complications)	Not adapted for deep-seated FCDs	

FCD, focal cortical dysplasia; SWE, shear wave elastography.

## Data Availability

Anonymized data will be shared on request from any qualified investigator.
